# Comparison of psychiatric emergency service admission rates in an italian COVID-19 hospital during lockdown and last year

**DOI:** 10.1192/j.eurpsy.2021.767

**Published:** 2021-08-13

**Authors:** F. Fiori Nastro, A. Mariano, R. Santini, F. Di Michele, F. Bianchi, C. Niolu

**Affiliations:** Department Of Systems Medicine, University of Rome “Tor Vergata”, Roma, Italy

**Keywords:** COVID-19, psychological impact, Hospital Admission rates, Psychiatric Emergency Service

## Abstract

**Introduction:**

Italy has been one of the most affected countries by Covid-19 pandemic. Our University General Hospital, Policlinico of “Tor Vergata” (PTV) in Rome, has become a Covid Hub in order to manage the spread of the epidemic. Thus, our Psychiatric Emergency Service (PES)’s access has been partially limited. Direct indicators of PES activity, as admission rates, can be useful for evaluating the psychological impact of Covid epidemic.

**Objectives:**

To assess psychiatric admittance rates to PES of PTV before and during Covid-19 global pandemic.

**Methods:**

Data from our PES register have been obtained and analyzed. We compared all the psychiatric access during the trimester March – May 2019 and 2020. All patients have been characterized according to clinical features.

**Results:**

A marked reduction of the number of patients presenting to PES has been observed (76 patients) in the 57-day period (March 11–May 04, 2020) of lockdown compared to the same period in 2019 (266 patients). The cutback was visible for all diagnostic groups, except for “Borderline Personality Disorder” diagnosis which have slightly increased. On the other hand, hospitalization rates in our psychiatric inpatients unit remained steady.

**Conclusions:**

Although larger study are needed to understand the mental consequences of the lockdown experience, people’s fear of potential infection might explain our results. Interestingly, personality disorder patients represent an exception to it, suggesting the importance of the clinical characteristics of fearless, engagement in dangerous behavior and detachment from reality. These findings might be helpful to improve psychosocial crisis interventions during the pandemic.

